# Supplementary material for: Determination of Parabens and Phenolic Compounds in Dairy Products through the Use of a Two-Step Continuous SPE System Including an Enhanced Matrix Removal Sorbent in Combination with UHPLC−MS/MS
Source: Foods. 2023 Jul 31;12(15):2909. doi: 10.3390/foods12152909 (PMC10418826; doi:10.3390/foods12152909)
Supplement: Supplementary file 1 [file foods-12-02909-s001.zip › foods-2508127-supplementary.pdf]

# Supplementary Material

## Determination of Parabens and Phenolic Compounds in Dairy Products through the Use of a Two-Step Continuous SPE System Including an Enhanced Matrix Removal Sorbent in Combination with UHPLC−MS/MS

**Laura Palacios Colón, Andrés J. Rascón and Evaristo Ballesteros ***

Department of Physical and Analytical Chemistry, E.P.S of Linares, University of Jaén, 23700 Linares, Jaén, Spain

**Table S1**. Results obtained for precision and recovery studies for the different types of dairy product samples.

| | | RSD (%) /Recovery (%) [a] | | | | | | | | | | | | | | | |
|---|---|---|---|---|---|---|---|---|---|---|---|---|---|---|---|---|---|
| | | Milk | | Yogurt | | Custard | | Milkshake | | Cream | | Cheese | | Butter | | Margarine | |
| | | **500 ng/kg** | **2000 ng/kg** | **500 ng/kg** | **2000 ng/kg** | **500 ng/kg** | **2000 ng/kg** | **500 ng/kg** | **2000 ng/kg** | **500 ng/kg** | **2000 ng/kg** | **500 ng/kg** | **2000 ng/kg** | **500 ng/kg** | **2000 ng/kg** | **500 ng/kg** | **2000 ng/kg** |
| **Parabens** | Methylparaben | 6.10[a] | 5.28 | 5.33 | 10.25 | 6.46 | 7.21 | 7.50 | 8.67 | 8.25 | 9.75 | 7.25 | 9.28 | 6.09 | 7.19 | 6.05 | 7.45 |
| | | 99 ± 10[a] | 101 ± 10 | 104 ± 7 | 101 ± 9 | 95 ± 7 | 95 ± 7 | 98 ± 10 | 101 ± 9 | 105 ± 9 | 102 ± 7 | 96 ± 11 | 93 ± 7 | 95 ± 6 | 102 ± 9 | 101 ± 6 | 106 ± 6 |
| | Ethylparaben | 9.20 | 7.16 | 6.30 | 11.40 | 5.39 | 8.08 | 7.86 | 7.27 | 5.85 | 7.98 | 5.69 | 7.35 | 6.44 | 8.91 | 5.52 | 8.51 |
| | | 101 ± 6 | 99 ± 10 | 91 ± 8 | 103 ± 8 | 105 ± 9 | 98 ± 5 | 103 ± 9 | 92 ± 6 | 91 ± 8 | 103 ± 9 | 101 ± 10 | 96 ± 8 | 101 ± 10 | 97 ± 6 | 101 ± 6 | 103 ± 7 |
| | Isopropylparaben | 5.28 | 7.35 | 7.55 | 9.87 | 6.80 | 9.39 | 5.33 | 9.73 | 8.47 | 9.50 | 9.05 | 5.52 | 8.34 | 11.46 | 6.00 | 10.37 |
| | | 103 ± 9 | 103 ± 10 | 93 ± 6 | 97 ± 7 | 98 ± 10 | 104 ± 8 | 95 ± 7 | 95 ± 7 | 94 ± 6 | 91 ± 9 | 97 ± 6 | 103 ± 9 | 103 ± 9 | 96 ± 9 | 102 ± 8 | 95 ± 9 |
| | Butylparaben | 5.36 | 7.83 | 8.05 | 7.84 | 7.21 | 9.47 | 6.46 | 6.27 | 5.48 | 9.58 | 9.38 | 9.17 | 5.69 | 10.81 | 5.49 | 10.44 |
| | | 98 ± 5 | 99 ± 9 | 104 ± 8 | 99 ± 10 | 98 ± 5 | 101 ± 6 | 99 ± 9 | 103 ± 9 | 95 ± 6 | 99 ± 9 | 104 ± 8 | 99 ± 9 | 102 ± 7 | 104 ± 8 | 99 ± 6 | 92 ± 7 |
| | Propylparaben | 8.01 | 9.56 | 6.21 | 10.57 | 7.18 | 9.87 | 8.80 | 6.20 | 7.87 | 7.38 | 11.07 | 8.54 | 6.64 | 6.58 | 6.62 | 9.50 |
| | | 104 ± 8 | 98 ± 10 | 104 ± 8 | 95 ± 6 | 101 ± 8 | 106 ± 6 | 102 ± 9 | 95 ± 6 | 104 ± 9 | 103 ± 9 | 96 ± 9 | 98 ± 5 | 96 ± 6 | 95 ± 6 | 102 ± 9 | 102 ± 7 |
| | Isobutylparaben | 8.29 | 7.30 | 9.66 | 10.22 | 7.37 | 8.42 | 5.77 | 8.81 | 8.48 | 10.14 | 7.10 | 6.54 | 6.49 | 7.26 | 8.59 | 10.60 |
| | | 102 ± 7 | 96 ± 8 | 94 ± 6 | 99 ± 9 | 104 ± 7 | 101 ± 6 | 97 ± 5 | 98 ± 9 | 99 ± 6 | 99 ± 10 | 104 ± 8 | 101 ± 6 | 101 ± 8 | 103 ± 8 | 99 ± 4 | 103 ± 10 |
| | Benzylparaben | 8.19 | 8.69 | 5.95 | 7.15 | 8.04 | 6.82 | 7.27 | 8.09 | 6.82 | 9.08 | 5.71 | 9.62 | 4.95 | 7.64 | 7.04 | 6.45 |
| | | 99 ± 7 | 104 ± 7 | 93 ± 6 | 102 ± 6 | 97 ± 6 | 98 ± 7 | 104 ± 9 | 101 ± 6 | 101 ± 9 | 104 ± 8 | 98 ± 10 | 104 ± 8 | 99 ± 10 | 100 ± 6 | 99 ± 8 | 98 ± 7 |

**Table S1**. Continued

| | | RSD (%) / Recovery (%)[a] | | | | | | | | | | | | | | | |
|---|---|---|---|---|---|---|---|---|---|---|---|---|---|---|---|---|---|
| | | Milk | | Yogurt | | Custard | | Milkshake | | Cream | | Cheese | | Butter | | Margarine | |
| | | 500 ng/kg | 2000 ng/kg | 500 ng/kg | 2000 ng/kg | 500 ng/kg | 2000 ng/kg | 500 ng/kg | 2000 ng/kg | 500 ng/kg | 2000 ng/kg | 500 ng/kg | 2000 ng/kg | 500 ng/kg | 2000 ng/kg | 500 ng/kg | 2000 ng/kg | 500 ng/kg | 2000 ng/kg |
| **Phenols** | Pentylphenol | 6.49[a] | 7.95 | 7.20 | 8.77 | 6.55 | 10.40 | 5.69 | 5.78 | 7.29 | 8.16 | 8.57 | 8.52 | 7.83 | 7.88 | 6.66 | 8.30 |
| | | 98 ± 6[a] | 99 ± 5 | 104 ± 8 | 97 ± 5 | 101 ± 6 | 99 ± 8 | 99 ± 8 | 97 ± 6 | 94 ± 7 | 104 ± 8 | 105 ± 10 | 98 ± 9 | 95 ± 7 | 100 ± 5 | 99 ± 8 | 105 ± 7 |
| | 4-Chloro-3-methylphenol | 5.29 | 9.56 | 5.20 | 5.27 | 10.67 | 4.65 | 9.38 | 9.55 | 6.10 | 10.54 | 8.48 | 5.34 | 10.15 | 6.19 | 9.15 | 6.89 |
| | | 104 ± 8 | 99 ± 9 | 105 ± 7 | 98 ± 9 | 98 ± 10 | 101 ± 9 | 91 ± 8 | 101 ± 10 | 93 ± 6 | 95 ± 7 | 92 ± 7 | 103 ± 5 | 99 ± 8 | 94 ± 7 | 103 ± 9 | 103 ± 9 |
| | 4-*tert*-Octylphenol | 5.39 | 8.49 | 5.65 | 4.34 | 7.46 | 9.50 | 4.20 | 10.47 | 5.40 | 10.21 | 8.45 | 8.33 | 5.75 | 8.49 | 3.59 | 6.57 |
| | | 99 ± 9 | 95 ± 8 | 99 ± 8 | 99 ± 8 | 103 ± 5 | 104 ± 8 | 102 ± 7 | 92 ± 6 | 94 ± 6 | 99 ± 9 | 94 ± 7 | 93 ± 6 | 103 ± 9 | 105 ± 9 | 98 ± 8 | 104 ± 9 |
| | Phenol | 8.08 | 8.37 | 5.95 | 7.91 | 9.57 | 10.34 | 6.14 | 8.21 | 7.24 | 11.30 | 6.52 | 7.53 | 3.08 | 6.56 | 8.88 | 3.25 |
| | | 104 ± 9 | 101 ± 6 | 101 ± 9 | 104 ± 8 | 98 ± 10 | 104 ± 8 | 103 ± 9 | 99 ± 6 | 98 ± 10 | 92 ± 7 | 105 ± 8 | 105 ± 7 | 98 ± 10 | 95 ± 7 | 100 ± 5 | 103 ± 5 |
| | 4-*tert*-Butylphenol | 5.59 | 11.1 | 5.44 | 6.36 | 7.39 | 9.34 | 5.29 | 9.65 | 6.27 | 9.40 | 9.86 | 8.40 | 7.51 | 9.82 | 7.01 | 9.40 |
| | | 96 ± 6 | 103 ± 6 | 102 ± 7 | 100 ± 8 | 99 ± 8 | 98 ± 5 | 101 ± 6 | 99 ± 9 | 103 ± 9 | 96 ± 9 | 98 ± 8 | 97 ± 9 | 96 ± 9 | 104 ± 8 | 102 ± 9 | 104 ± 8 |
| | Nonylphenol | 7.64 | 10.31 | 5.84 | 9.24 | 6.65 | 7.54 | 5.55 | 9.37 | 5.32 | 9.81 | 10.77 | 9.25 | 5.58 | 10.74 | 6.08 | 7.17 |
| | | 98 ± 10 | 101 ± 9 | 96 ± 11 | 101 ± 9 | 101 ± 6 | 99 ± 9 | 103 ± 9 | 103 ± 9 | 101 ± 10 | 104 ± 7 | 93 ± 6 | 102 ± 6 | 97 ± 6 | 101 ± 9 | 95 ± 6 | 91 ± 8 |
| | 2-*tert*-Butylphenol | 5.70 | 10.44 | 7.02 | 9.41 | 7.57 | 10.51 | 7.08 | 6.17 | 6.22 | 9.61 | 6.38 | 9.34 | 5.78 | 9.50 | 7.24 | 8.97 |
| | | 99 ± 8 | 102 ± 6 | 102 ± 7 | 99 ± 9 | 98 ± 10 | 95 ± 7 | 100 ± 5 | 96 ± 9 | 98 ± 5 | 99 ± 9 | 104 ± 8 | 95 ± 6 | 101 ± 8 | 106 ± 6 | 99 ± 9 | 101 ± 9 |
| | Bisphenol F | 5.99 | 9.55 | 6.04 | 8.27 | 9.41 | 8.24 | 5.62 | 8.3 | 5.89 | 7.91 | 6.50 | 6.57 | 6.65 | 6.24 | 5.34 | 11.31 |
| | | 101 ± 6 | 94 ± 5 | 91 ± 8 | 101 ± 10 | 93 ± 6 | 93 ± 7 | 104 ± 8 | 103 ± 5 | 100 ± 5 | 103 ± 9 | 94 ± 7 | 103 ± 9 | 101 ± 10 | 96 ± 11 | 102 ± 7 | 104 ± 8 |
| | 3,4-Dimehylphenol | 7.00 | 10.64 | 4.29 | 9.61 | 8.25 | 9.35 | 5.77 | 9.58 | 5.56 | 8.36 | 6.52 | 6.40 | 8.28 | 6.49 | 6.24 | 10.81 |
| | | 100 ± 5 | 101 ± 9 | 95 ± 7 | 98 ± 10 | 97 ± 5 | 104 ± 8 | 97 ± 5 | 101 ± 6 | 93 ± 6 | 104 ± 8 | 98 ± 10 | 98 ± 5 | 99 ± 8 | 105 ± 7 | 103 ± 9 | 103 ± 9 |
| | 2,5-Dimethylphenol | 6.69 | 7.71 | 5.51 | 9.32 | 9.09 | 10.71 | 9.81 | 7.16 | 5.44 | 10.64 | 5.67 | 9.38 | 6.43 | 6.78 | 7.60 | 6.38 |
| | | 100 ± 8 | 99 ± 8 | 98 ± 5 | 95 ± 7 | 101 ± 6 | 106 ± 6 | 103 ± 9 | 100 ± 8 | 105 ± 7 | 91 ± 6 | 103 ± 6 | 103 ± 9 | 97 ± 8 | 104 ± 8 | 91 ± 9 | 95 ± 6 |
| | 4-Phenylphenol | 5.33 | 10.13 | 6.15 | 9.19 | 11.30 | 8.56 | 8.03 | 7.23 | 6.32 | 10.98 | 6.76 | 5.45 | 5.29 | 7.25 | 5.47 | 8.09 |
| | | 99 ± 5 | 93 ± 6 | 100 ± 5 | 94 ± 7 | 100 ± 8 | 91 ± 8 | 102 ± 7 | 96 ± 8 | 97 ± 6 | 97 ± 5 | 95 ± 10 | 100 ± 5 | 93 ± 6 | 100 ± 5 | 93 ± 6 | 103 ± 8 |
| | 2-Phenylphenol | 6.32 | 9.25 | 7.25 | 11.30 | 6.17 | 7.26 | 9.03 | 6.24 | 5.25 | 11.01 | 5.68 | 7.55 | 8.98 | 9.41 | 5.55 | 7.56 |
| | | 96 ± 6 | 93 ± 6 | 102 ± 7 | 104 ± 6 | 101 ± 9 | 104 ± 8 | 98 ± 5 | 103 ± 9 | 99 ± 4 | 101 ± 10 | 100 ± 5 | 93 ± 6 | 100 ± 5 | 91 ± 9 | 101 ± 6 | 104 ± 7 |
| | Bisphenol S | 4.35 | 10.61 | 8.26 | 9.41 | 6.19 | 9.45 | 7.58 | 6.19 | 8.94 | 7.98 | 8.32 | 5.44 | 6.34 | 6.47 | 7.18 | 9.41 |
| | | 103 ± 9 | 104 ± 8 | 103 ± 9 | 105 ± 8 | 102 ± 8 | 99 ± 8 | 101 ± 10 | 101 ± 9 | 91 ± 9 | 99 ± 9 | 95 ± 8 | 99 ± 8 | 105 ± 9 | 95 ± 8 | 99 ± 8 | 99 ± 8 |
| | 4-Chlorophenol | 6.28 | 10.90 | 5.24 | 10.75 | 6.35 | 9.30 | 7.14 | 6.30 | 5.99 | 6.35 | 7.57 | 9.31 | 5.56 | 7.41 | 6.55 | 8.20 |
| | | 102 ± 7 | 98 ± 5 | 102 ± 6 | 104 ± 7 | 105 ± 7 | 96 ± 8 | 96 ± 6 | 102 ± 9 | 98 ± 5 | 105 ± 7 | 99 ± 9 | 104 ± 8 | 99 ± 9 | 98 ± 5 | 101 ± 10 | 101 ± 9 |
| | 4-Hexylphenol | 9.09 | 11.09 | 6.46 | 5.28 | 8.33 | 9.41 | 6.08 | 6.39 | 5.31 | 9.35 | 6.15 | 10.41 | 7.74 | 10.61 | 5.35 | 9.08 |
| | | 103 ± 9 | 102 ± 6 | 98 ± 5 | 99 ± 9 | 91 ± 9 | 94 ± 7 | 105 ± 7 | 98 ± 10 | 105 ± 7 | 99 ± 10 | 95 ± 7 | 100 ± 5 | 100 ± 7 | 93 ± 6 | 100 ± 5 | 94 ± 7 |
| | Bisphenol A | 5.34 | 8.42 | 6.86 | 6.28 | 7.56 | 10.81 | 9.83 | 9.83 | 5.91 | 9.89 | 7.07 | 10.50 | 7.64 | 9.60 | 7.21 | 11.38 |
| | | 98 ± 8 | 99 ± 8 | 101 ± 10 | 101 ± 9 | 91 ± 9 | 98 ± 5 | 93 ± 7 | 94 ± 7 | 97 ± 7 | 98 ± 5 | 100 ± 5 | 93 ± 6 | 100 ± 5 | 96 ± 6 | 103 ± 6 | 102 ± 7 |
| | Bisphenol B | 6.63 | 7.20 | 5.68 | 5.55 | 10.45 | 8.27 | 6.35 | 8.27 | 5.42 | 9.60 | 6.70 | 9.51 | 5.94 | 8.20 | 6.25 | 9.40 |
| | | 103 ± 5 | 101 ± 6 | 99 ± 9 | 98 ± 8 | 103 ± 7 | 103 ± 8 | 96 ± 11 | 96 ± 11 | 105 ± 6 | 105 ± 8 | 102 ± 8 | 96 ± 7 | 102 ± 7 | 94 ± 6 | 91 ± 8 | 92 ± 7 |
| | 4-Heptylphenol | 6.35 | 8.47 | 5.39 | 7.22 | 6.98 | 6.61 | 4.68 | 9.35 | 8.91 | 9.30 | 5.25 | 10.34 | 5.84 | 9.37 | 6.80 | 7.44 |
| | | 101 ± 9 | 91 ± 8 | 103 ± 9 | 99 ± 8 | 104 ± 7 | 93 ± 6 | 100 ± 7 | 105 ± 8 | 98 ± 5 | 105 ± 7 | 91 ± 8 | 101 ± 9 | 106 ± 6 | 98 ± 5 | 96 ± 7 | 95 ± 6 |
| | Bisphenol Z | 6.62 | 9.55 | 6.28 | 8.15 | 7.19 | 8.45 | 5.85 | 6.78 | 6.13 | 7.24 | 7.34 | 9.35 | 9.82 | 8.50 | 5.29 | 11.32 |
| | | 98 ± 10 | 99 ± 9 | 99 ± 4 | 103 ± 10 | 105 ± 8 | 92 ± 5 | 91 ± 6 | 103 ± 6 | 106 ± 6 | 103 ± 9 | 100 ± 8 | 94 ± 7 | 98 ± 8 | 101 ± 10 | 91 ± 8 | 91 ± 8 |
| | Pentachlorophenol | 8.85 | 7.75 | 7.41 | 6.88 | 8.65 | 6.30 | 10.32 | 8.15 | 7.17 | 7.48 | 7.91 | 11.02 | 5.82 | 8.36 | 6.38 | 10.94 |
| | | 105 ± 6 | 95 ± 8 | 91 ± 9 | 94 ± 7 | 105 ± 7 | 104 ± 7 | 105 ± 7 | 101 ± 6 | 106 ± 6 | 93 ± 6 | 100 ± 9 | 101 ± 10 | 98 ± 7 | 93 ± 6 | 99 ± 9 | 96 ± 8 |
| | Triclosan | 9.13 | 5.64 | 8.23 | 6.13 | 7.18 | 9.40 | 8.07 | 7.50 | 5.55 | 10.10 | 6.16 | 7.22 | 8.15 | 10.13 | 5.24 | 5.78 |
| | | 94 ± 7 | 105 ± 7 | 105 ± 7 | 98 ± 10 | 97 ± 8 | 105 ± 6 | 96 ± 9 | 98 ± 6 | 104 ± 9 | 98 ± 8 | 103 ± 9 | 102 ± 6 | 104 ± 8 | 93 ± 7 | 105 ± 8 | 99 ± 8 |

[a] RSD: relative standard deviation (n=12) for 500 and 2000 ng/kg ($p < 0.05$), interday. Percent recoveries (% ± SD, n = 3) of analytes spiked to milk and dairy product samples (500 – 2 000 ng/L)

**Table S2.** Results obtained in the matrix effect studies of the different types of dairy product samples

| | | **Matrix Effect (%)[a]** | | | | | | | |
|---|---|---|---|---|---|---|---|---|---|
| | | Milk | Yoghourt | Cheese | Milkshake | Cream | Custard | Butter | Margarine |
| **Parabens** | Methylparaben | 1.03 (3%) | 0.98 (-2%) | 0.94 (-6%) | 1.06 (6%) | 0.95 (-5%) | 0.97 (-3%) | 1.02 (2%) | 0.92 (-7%) |
| | Ethylparaben | 0.98 (-2%) | 1.04 (4%) | 1.05 (5%) | 0.93 (-7%) | 0.99 (-1%) | 1.03 (3%) | 0.95 (-5%) | 0.93 (-7%) |
| | Isopropylparaben | 1.07 (7%) | 0.98 (-2%) | 0.94 (-6%) | 1.05 (5%) | 0.95 (-5%) | 1.03 (3%) | 1.05 (5%) | 0.93 (-7%) |
| | Butylparaben | 1.02 (2%) | 0.92 (-8%) | 0.99 (-1%) | 1.01 (1%) | 0.95 (-5%) | 0.90 (-10%) | 1.01 (1%) | 1.02 (2%) |
| | Propylparaben | 1.02 (2%) | 0.92 (-7%) | 0.95 (-5%) | 0.90 (-10%) | 1.06 (6%) | 0.92 (-8%) | 0.90 (-10%) | 0.98 (-2%) |
| | Isobutylparaben | 0.95 (-5%) | 0.93 (-7%) | 0.93 (-7%) | 1.04 (4%) | 0.93 (-7%) | 1.04 (4%) | 1.04 (4%) | 0.99 (-1%) |
| | Benzylparaben | 0.99 (-1%) | 1.05 (5%) | 1.03 (3%) | 0.98 (-2%) | 1.03 (3%) | 0.98 (-2%) | 0.99 (-1%) | 1.05 (5%) |
| **Phenols** | Pentylphenol | 1.02 (2%) | 0.95 (-5%) | 0.90 (-10%) | 0.98 (-2%) | 1.02 (2%) | 0.92 (-7%) | 0.94 (-6%) | 1.04 (4%) |
| | 4-Chloro-3-methylphenol | 1.07 (7%) | 1.06 (6%) | 0.92 (-8%) | 0.93 (-7%) | 0.93 (-7%) | 0.97 (-3%) | 0.90 (-10%) | 1.04 (4%) |
| | 4-*tert*-Octylphenol | 0.95 (-5%) | 0.93 (-7%) | 0.95 (-5%) | 0.93 (-7%) | 1.02 (2%) | 0.93 (-7%) | 0.95 (-5%) | 0.93 (-7%) |
| | Phenol | 0.99 (-1%) | 1.03 (3%) | 0.95 (-5%) | 1.04 (4%) | 1.05 (5%) | 1.06 (6%) | 1.04 (4%) | 1.02 (2%) |
| | 4-*tert*-Butylphenol | 0.95 (-5%) | 1.03 (3%) | 0.90 (-10%) | 0.92 (-7%) | 0.96 (-4%) | 0.95 (-5%) | 0.98 (-2%) | 0.99 (-1%) |
| | Nonylphenol | 0.98 (-2%) | 0.93 (-7%) | 0.97 (-3%) | 0.93 (-7%) | 0.94 (-6%) | 1.03 (3%) | 0.99 (-1%) | 1.03 (3%) |
| | 2-*tert*-Butylphenol | 0.98 (-2%) | 1.02 (2%) | 0.93 (-7%) | 1.02 (2%) | 0.93 (-7%) | 0.93 (-7%) | 0.93 (-7%) | 1.06 (6%) |
| | Bisphenol F | 0.94 (-6%) | 1.03 (3%) | 0.99 (-1%) | 1.03 (3%) | 0.99 (-1%) | 0.93 (-7%) | 1.02 (2%) | 1.10 (10%) |
| | 3,4-Dimethylphenol | 0.93 (-7%) | 1.06 (6%) | 1.02 (2%) | 0.92 (-7%) | 1.02 (2%) | 0.93 (-7%) | 0.92 (-8%) | 0.92 (-8%) |
| | 2,5-Dimethylphenol | 0.98 (-2%) | 0.92 (-7%) | 0.93 (-7%) | 1.04 (4%) | 0.90 (-10%) | 0.98 (-2%) | 0.93 (-7%) | 0.95 (-5%) |
| | 4-Phenylphenol | 0.95 (-5%) | 1.04 (4%) | 0.90 (-10%) | 1.04 (4%) | 1.02 (2%) | 0.94 (-6%) | 1.06 (6%) | 0.95 (-5%) |
| | 2-Phenylphenol | 1.01 (1%) | 1.04 (4%) | 0.95 (-5%) | 0.93 (-7%) | 0.99 (-1%) | 1.05 (5%) | 1.05 (5%) | 0.93 (-7%) |
| | Bisphenol S | 0.99 (-1%) | 0.93 (-7%) | 1.05 (5%) | 1.06 (6%) | 0.98 (-2%) | 0.92 (-7%) | 1.06 (6%) | 0.93 (-7%) |
| | 4-Chlorophenol | 1.04 (4%) | 1.02 (2%) | 0.96 (-4%) | 0.95 (-5%) | 0.99 (-1%) | 1.05 (5%) | 0.95 (-5%) | 1.02 (2%) |
| | 4-Hexylphenol | 1.05 (5%) | 1.01 (1%) | 1.06 (6%) | 0.96 (-6%) | 0.92 (-8%) | 1.02 (2%) | 0.93 (-7%) | 0.99 (-1%) |
| | Bisphenol A | 1.02 (2%) | 0.94 (-6%) | 1.04 (4%) | 1.01 (1%) | 1.06 (6%) | 0.98 (-2%) | 1.08 (8%) | 0.95 (-5%) |
| | Bisphenol B | 0.99 (-1%) | 1.05 (5%) | 0.95 (-5%) | 0.99 (-1%) | 0.95 (-5%) | 0.99 (-1%) | 1.02 (2%) | 0.95 (-5%) |
| | 4-Heptylphenol | 0.94 (-6%) | 1.04 (4%) | 1.05 (5%) | 1.04 (4%) | 0.98 (-2%) | 1.02 (2%) | 0.93 (-7%) | 1.04 (4%) |
| | Bisphenol Z | 0.92 (8%) | 1.01 (1%) | 1.08 (8%) | 0.92 (-8%) | 1.08 (8%) | 1.05 (5%) | 0.92 (-8%) | 0.93 (-7%) |
| | Pentachlorophenol | 0.90 (-10%) | 1.02 (2%) | 1.08 (8%) | 1.02 (2%) | 0.91 (-9%) | 0.98 (-2%) | 0.92 (-7%) | 0.95 (-5%) |
| | Triclosan | 1.02 (2%) | 1.06 (6%) | 0.91 (-9%) | 1.06 (6%) | 1.01 (1%) | 0.93 (-7%) | 1.06 (6%) | 1.06 (6%) |

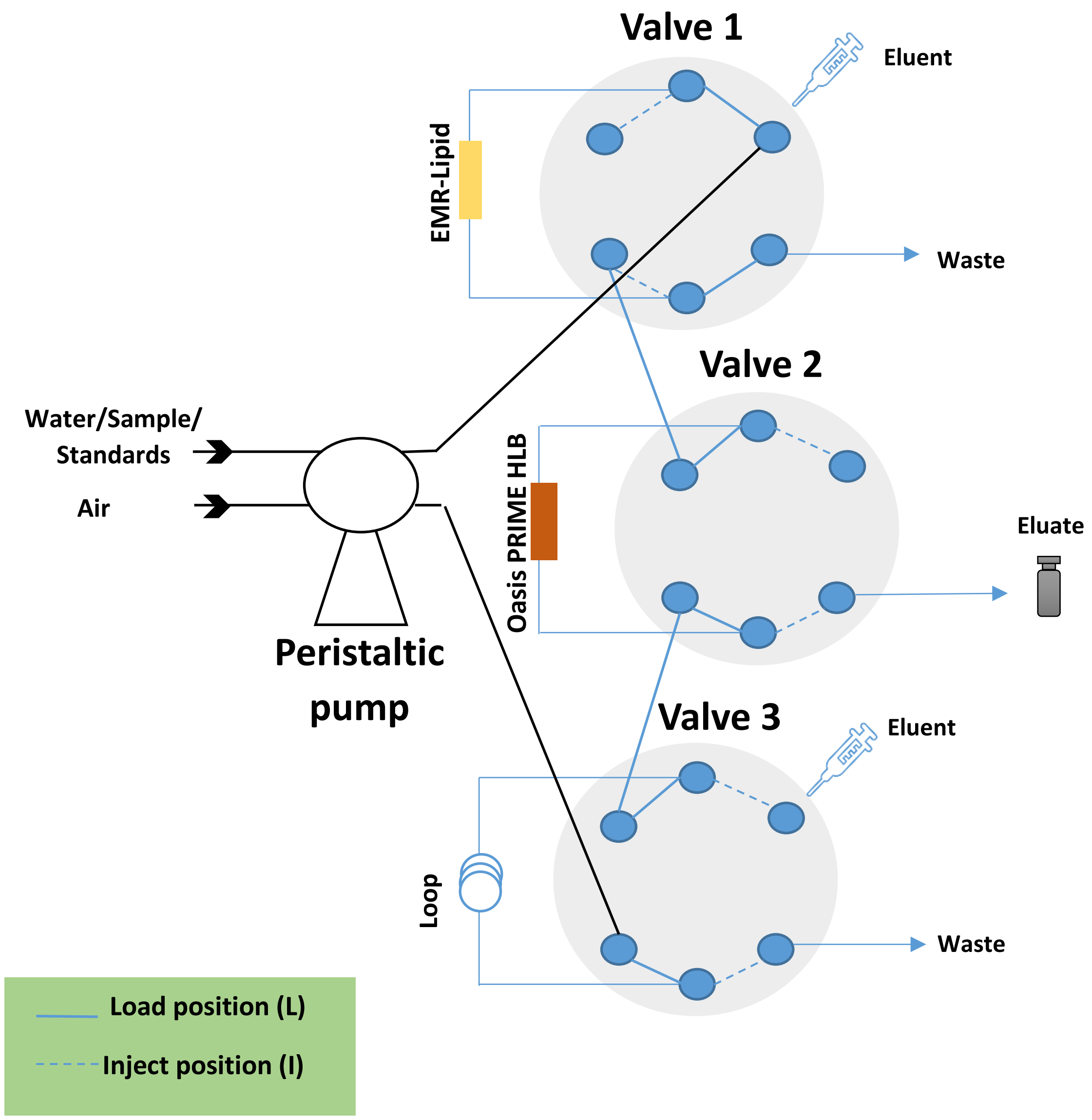


| Step | Valve 1 | Valve 2 | Valve 3 |
|---|---|---|---|
| 1. ERM-Lipid sorbent conditioning | L | I | I |
| 2. Oasis PRiME HLB sorbent conditioning | I | L | I |
| 3. Introduction of sample or standards | L | L | L |
| 4. Oasis PRiME HLB drying | L | I | L |
| 5. Elution of analytes | L | I | I |

Figure S1. Continuous-flow system for the solid-phase with three injection valves and two serially columns.

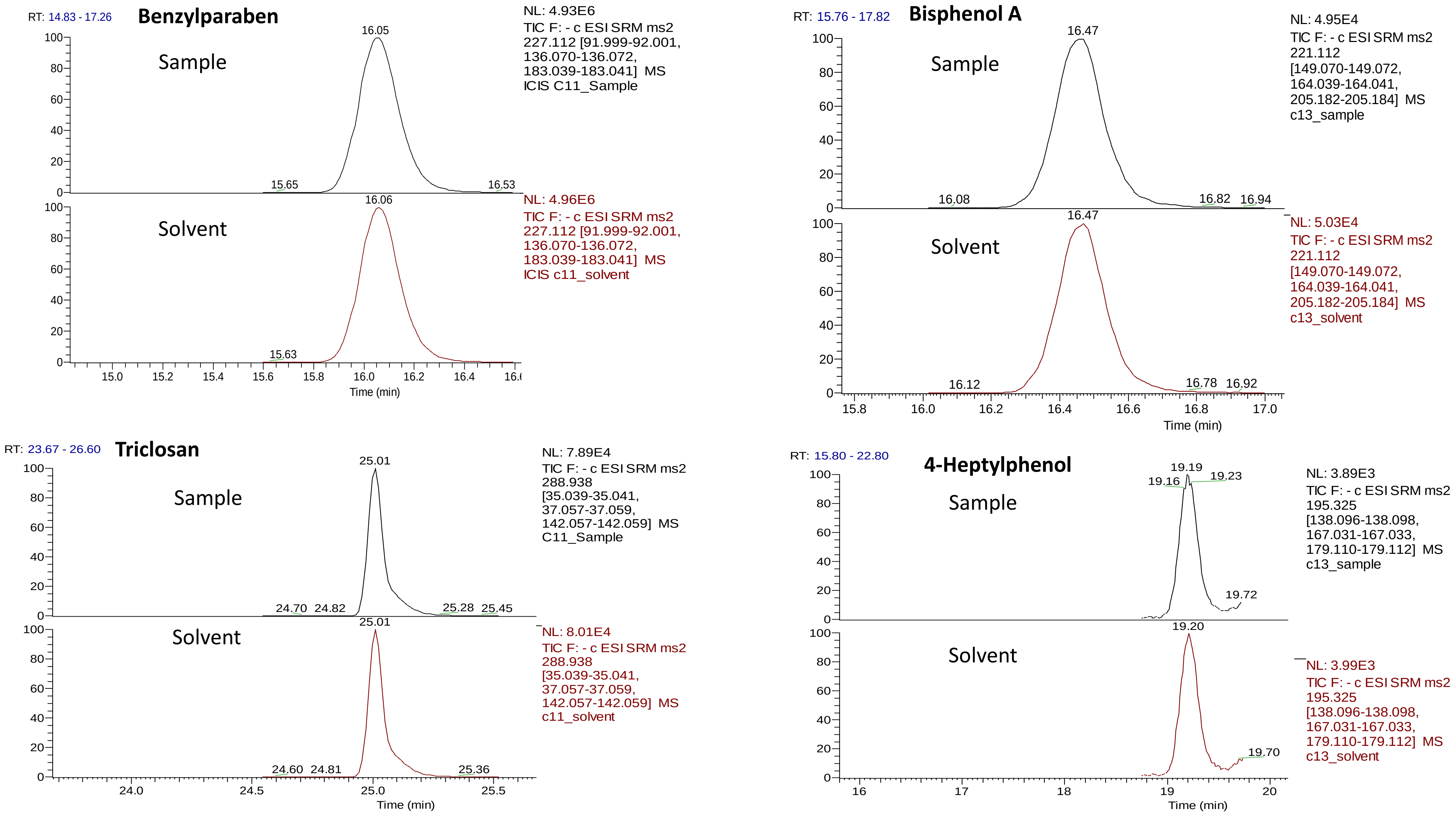


Figure S2. Extract ion chromatograms for four analytes (benzylparaben, bisphenol A, triclosan and 4-heptylphenol). In one chromatogram, the analyte is spiked in the solvent, and in the other the analyte is spiked in a milk sample.
